# Can digital twin technology revolutionize public health emergency management? Insights from the PPRR framework

**DOI:** 10.3389/fpubh.2025.1631339

**Published:** 2025-09-25

**Authors:** Tianze Sun, Jialin Lu, Qicheng Li, Yuhui Kou

**Affiliations:** ^1^Department of Trauma and Orthopedics, Peking University People's Hospital, Beijing, China; ^2^Key Laboratory of Trauma and Neural Regeneration (Peking University), Ministry of Education, Beijing, China; ^3^Department of Health Policy, School of Health Management, Harbin Medical University, Harbin, China; ^4^National Center for Trauma Medicine, Beijing, China

**Keywords:** digital twin, public health, emergency, PPRR, management

## Abstract

The inherent nature of public health emergencies often renders them unpredictable and inadequately managed in a timely manner, resulting in significant impacts on lives, property, and social stability. In recent years, the frequency and severity of such emergencies have escalated. As an integration of artificial intelligence, machine learning, deep learning, big data, and the Internet of Things, digital twin technology has been increasingly adopted across diverse fields, including industrial production, urban planning, and healthcare. This study aimed to investigate recent advancements in digital twin technology, particularly in the fields of medicine and urban construction, with a focus on its potential to mitigate the impacts of public health crises. By employing the PPRR framework, this study further explored the prospects for constructing and applying digital twin technology within the public health management system. Taking perceived fidelity (SF), model plasticity (MP), decision coupling (DC), and governance credibility (GC) as the core constructs, we discussed the operability of unified modeling and quantitative evaluation of each stage of digital twin implementation. This approach comprehensively explained all four phases of emergency management: prevention, preparedness, response, and recovery. Through enabling the digitalization, intelligence, and visualization of the entire health emergency management process, digital twin technology offers holistic solutions throughout the life cycle of public health emergencies. These include preventive measures, real-time detection, early warning systems, and intelligent emergency response management. Such innovations have the potential to significantly enhance management efficiency and effectiveness, bolster coordinated responses to public health crises, and improve the overall resilience of public health systems.

## 1 Introduction

Public health emergencies are sudden, urgent, and unpredictable events that present significant challenges in terms of rapid identification, response, and treatment. These emergencies not only endanger the safety of citizens’ lives and property but also have the potential to incite public panic and cause substantial socio-economic losses (1, 2). The emergency response framework for public health crises seeks to establish a systematic and well-coordinated response system to effectively prevent, respond to, and mitigate the impact of such events (3, 4). It is noteworthy that the COVID-19 global pandemic, as the most severe public health crisis of this century, not only tested the core value of the existing emergency response system, but also exposed many structural deficiencies (5, 6). In particular, the rigid structure and data silo phenomenon of the public health information system severely limited the accuracy of epidemic investigation and assessment and the effectiveness of resource deployment (7, 8). These lessons highlight the urgent need to transform and upgrade the traditional emergency management model to a more resilient, intelligent prevention and control system in the context of the emergence of new infectious diseases. The limitations of existing structures—such as delayed responses and operational dysfunction—were exacerbated, resulting in a lack of proactive, coherent, and logical response strategies (6). In light of these challenges, there is a clear need for a more effective system capable of swiftly responding to major public health emergencies, providing real-time, continuous monitoring and prediction of health threats, and controlling the spread of infectious diseases. This would not only reduce the likelihood of adverse events but also enhance the active feedback and response mechanisms, ultimately improving the overall capacity and efficiency of public health emergency management (9, 10).

The concept of Digital Twin (DT) was originally introduced to describe product lifecycle management, encompassing physical products, virtual models, and their interconnections (11, 12). Research on DT has surged in recent years, positioning it among Gartner’s top 10 strategic technology trends for 2019. It is projected that by 2027, over 40% of large organizations worldwide will integrate Web3, spatial computing, and digital twins in metaverse-based projects (13). As an engineering concept that mirrors the physical world through virtual replicas, DT bridges the gap between the physical and virtual realms. This connection enables real-time monitoring, design thinking, visualization, preventive maintenance, design verification, and operational optimization (14). As a result, digital twin technology has been successfully applied in diverse fields, including workshop management, intelligent manufacturing, and urban development (15–17). When digital twins are redefined as virtual representations of both living and non-living entities, they open new possibilities for applications in human and public health (18, 19). Early-stage implementations have already been seen in the medical sector, such as the creation of healthcare data, digital representations of hospital environments and surgical operations, research on virtual human models, simulations predicting patient responses to drugs, and the establishment of digital twin hospitals and digital health communities (20–22).

Digital twin technology offers a novel framework for advancing personalized medicine by leveraging personalized data and optimizing multi-source integrated clinical strategies (23). It also holds significant potential for enhancing the emergency management of public health events. By creating virtual models of physical entities and utilizing real-time data alongside algorithmic models, the evolving dynamics of major events can be analyzed and identified. This enables real-time responses, effective control, and accurate predictions, facilitating the integration of existing social resources and strengthening the capacity to manage public health emergencies (24, 25). Building on the PPRR (Prevention, Preparedness, Response, and Recovery) framework, we propose the development of a public health management system centered around digital twin technology. The DT-PPRR fusion paradigm, which takes perceived fidelity (SF), model plasticity (MP), decision coupling (DC) and governance credibility (GC) as the core constructs to analyze the role of digital twins in prevention, preparation, response and recovery (26–29). Conduct unified modeling and quantitative evaluation of value realization at each stage to form a testable theoretical framework. Through collaboration between the healthcare sector, the artificial intelligence industry, and relevant government agencies, we can continuously monitor the progression of epidemics and provide strategic guidance for the prevention and control of public health crises. By offering timely information and recommendations for decision-making, this approach is expected to play a pivotal role in future epidemic prevention, resource management, and decision support, ultimately contributing to global public health security (30, 31).

## 2 Theoretical basis

### 2.1 PPRR theory

The PPRR (Prevention, Preparedness, Response, and Recovery) theory is a crucial theoretical framework in the field of emergency management (32). Originating in the United States, it has gained widespread adoption in research related to health and public health emergencies in recent years (33). This theoretical model encompasses four interconnected stages: Prevention, Preparedness, Response, and Recovery. These stages form a continuous loop through their interactions, effectively supporting the digital and intelligent processes involved in the prevention and management of public health emergencies.

**Prevention:** This stage involves implementing measures to prevent the occurrence of adverse events. The goal is to protect people’s lives, health, and property by reducing or eliminating risk factors before an event can take place.

**Preparedness:** The focus of this stage is ensuring that the resources, capabilities, and systems necessary to respond to public health incidents or disasters are in place and readily available. The objective is to enhance the efficiency and effectiveness of emergency responses and to minimize confusion and panic when incidents occur.

**Response:** In this stage, immediate actions are taken when a public health emergency arises to mitigate its adverse effects and protect lives and property. The purpose is to control the development of the incident and minimize its impact.

**Recovery:** This phase involves the process of restoring society, the economy, the environment, and public life to normal or improved conditions once the impact of the public health emergency has been largely controlled. The goal is to derive lessons from the emergency to improve the overall emergency management capabilities of the health system in the future.

### 2.2 Digital twin

The concept of the digital twin was first introduced by Professor Grieves for product lifecycle management (34, 35). It refers to the accurate virtual representation of physical objects, processes, or systems within a digital space (36). This high-level simulation integrates historical data and real-time sensor data, encompassing both the physical and functional characteristics of real-world models (37, 38). Digital twins serve as in silico copies of physical entities, utilizing multimodal data to build models that optimize decision-making and predict the effects of interventions on outcomes. This operable data connection system between the virtual and physical realms collects real-time data from physical objects and transmits it to their digital counterparts for processing and analysis, thereby providing precise analytical insights and simulating potential scenarios (39). The digital twin transforms the performance and condition of real-world objects in the physical environment. Initially, the three-dimensional model of a digital twin consisted of physical products, virtual representations, and their interconnections (40). As digital technologies advanced and the concept of digital twins gained traction among experts and scholars, the five-dimensional model of digital twins was proposed (41). This expanded model includes physical entities, virtual models, services, twin data, and the connections between these components. The core idea of this model is to establish real-time interactive feedback between physical entities and their digital counterparts through data transmission, enabling real-time monitoring, simulation analysis, and predictive optimization. This allows for the full-cycle monitoring, management, and optimization of an object or system.

Digital twins fulfill the need for real-time, two-way connections and interaction by integrating various information technologies such as the Internet of Things (IoT), big data, artificial intelligence (AI), and deep learning (42). IoT is a key technology to strengthen digital transformation, and it involves a broad system of interconnected “things.” IoT collect and link data collected by sensors and other devices, using edge computing to perform preliminary processing close to the site, and performing aggregation and recalculation in the cloud (43). This determines the freshness and scalability of data-to-model and is the technical basis for the real-time nature of digital twins. Blockchain is a decentralized and growing chain of records, which uses distributed ledgers to record the “who used it, when and why” of data, and has the characteristics of non-tampering and traceability (43, 44). These technologies enable both virtual and physical realms to interact seamlessly, creating a robust platform for real-time data exchange. System-level and complex system-level digital twins can dynamically address diverse computing, storage, and operational requirements through on-demand cloud computing services. Additionally, 5G communication technology, with its high speed, large capacity, and low latency, supports the data transmission and equipment interconnection essential for digital twins (45). This facilitates real-time data exchange between virtual models and physical entities, thereby accelerating the application of digital twin technology. Twin data integrates multi-source, multi-type, and multi-structured data, often encompassing vast volumes of information (46, 47). To manage big data effectively, a comprehensive database can be established to extract valuable insights that explain and predict the behaviors and outcomes of the physical world (48). Blockchain technology further strengthens the reliability and security of digital twins by ensuring data integrity and providing traceability throughout the process. The trust mechanism enabled by blockchain ensures that users can confidently access and utilize various services (49). Furthermore, artificial intelligence enhances the value of twin data by automatically conducting data analysis and deep learning, utilizing optimal algorithms to improve the responsiveness and accuracy of services.

## 3 Application examples of digital twins

### 3.1 Application of digital twins in the medical field

Although digital twin is recognized as an advanced simulation architecture in the field of engineering technology, its development potential in the field of healthcare cannot be ignored. Due to the variety of ways to obtain patient data, the incomplete mechanism model of disease pathophysiology, and the interference of factors outside biological constraints, the “passive” data collection of traditional digital medicine is based on the data of a large number of retrospectively scanned models to predict future health conditions, while lacking “active” feedback from patients. Digital twins can and leverage continuous, real-time data streams to reactively update predictions, providing an opportunity to broaden the scope of medical digital twins.

**Figure 1:**
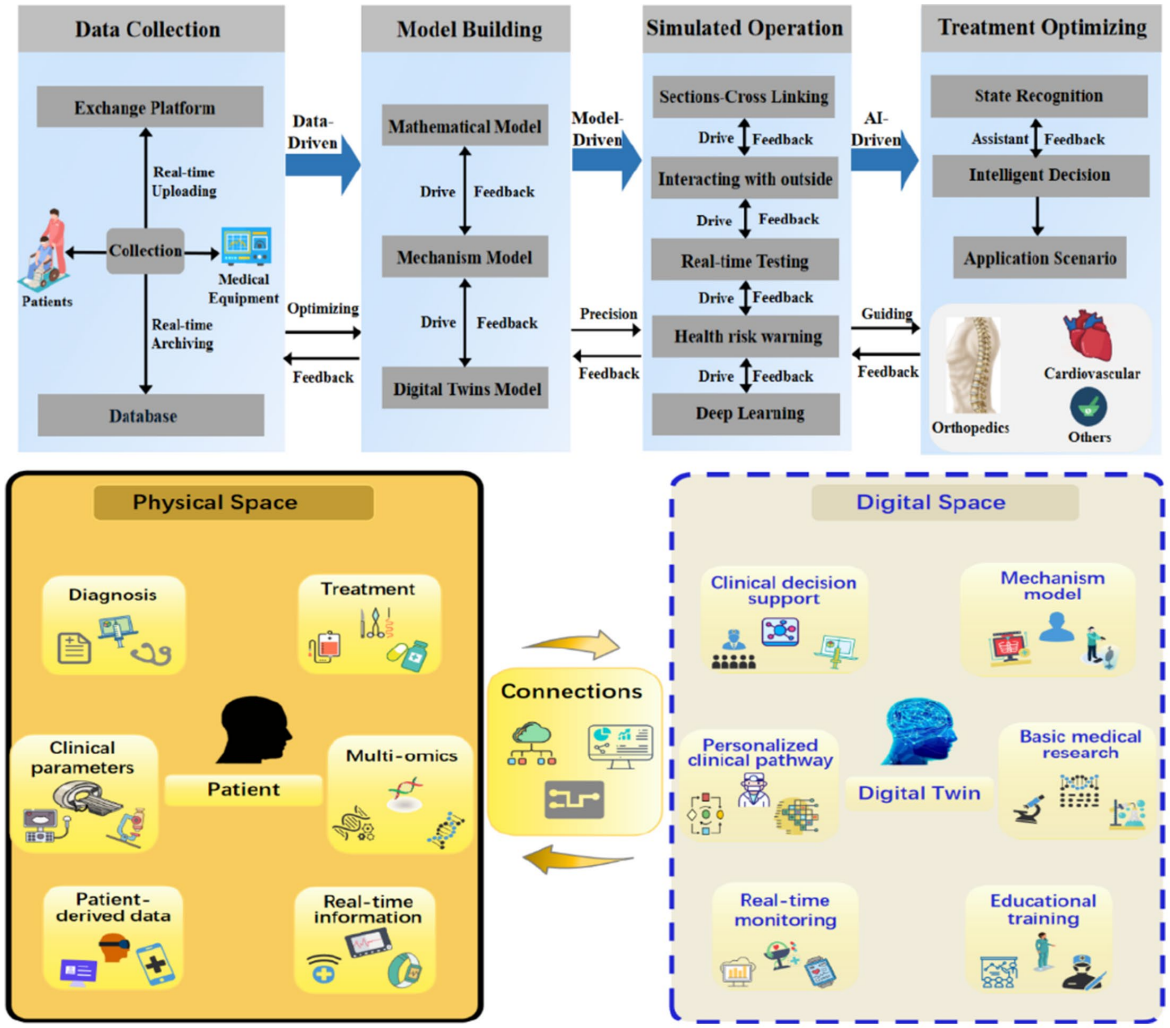
Application paradigm of digital twin technology in the medical field (23).

In recent years, with the development of personalized and precision medicine, the application of digital twins in the healthcare sector has garnered increasing attention (23). Researchers can create a virtual replica of a patient or an organ in a virtual space, using various Internet technologies to construct a digital twin and perform planned operations on the replica, observing the real-time feedback to optimize the treatment process and select the most effective treatment methods. Figure 1 depicted the construction process and application diagram of digital twins in the medical field. The process of digital twin construction included data perception and collection, model construction, simulation exercises and optimization in the actual treatment process. Considering the characteristics of the digital twin itself, its construction process and application scenarios collect patient data such as diagnosis, treatment, clinical examination parameters, and clinical symptoms in physical space. After the calculation and optimization of the big data model, real-time optimization schemes and decision-making suggestions were provided for doctors’ clinical decisions, patients’ clinical pathways, basic research and mechanism model construction in digital space. Table 1 summarizes the typical applications of digital twin technology in the medical field in recent years.

**Table 1:** Representative applications of DT in medical fields.

Year	Team	Applications of DT in medical field	The characteristics of DTs in medical field
2011	Niederer et al. (51)	Using mechanical models to investigate the dependence of the CRT efficacy on cellular-scale mechanisms and organs.	It was used to predicts that a patient will respond less to CRT treatment and identify novel patient selection criteria.
2014	Baillargeon et al. (60)	The project developed a DT model simulating the human heart to view interactions of an organ with medicines.	It could help doctors and pharmaceutical engineers can see the complex structure or the mobility of heart tissue.
2018	Prakosa et al. (52)	They used DT technology in the field of infarct-related ventricular tachycardia.	It improved the ablation guidance through the identification of patient-specific optimal targets before a clinical procedure.
2018	The Philips (50)	The Philips Heart Navigator tool combines CT images in a single image of the patient’s heart anatomy.	It provides real-time 3D insight into the positioning of devices during surgery, which can simplify the prior procedure planning.
2019	Chakshu et al. (56)	They built a DT model coupled with blood flow and head vibration to develop diagnostic tools.	Comparing the *in vivo* vibration against the virtual data to detect the severity of carotid stenosis from a video of a human face.
2020	Mussomeli et al. (61)	Takeda Pharmaceuticals switched to DT technology in production to offer transformative therapies around the world.	DT models could shorten pharmaceutical processes and allow realistic input–output predictions of biochemical reactions.
2020	Erol et al. (62)	Atos and Siemens worked with the pharmaceutical industry to establish DT models supported by the IoT, artificial intelligence and many other advanced technologies.	It improved the manufacturing process using physical DT models, which were created to overcome difficulties in terms of efficiency and production
2020	Shamanna et al. (71)	They established a DT body based on daily continuous glucose monitor and food intake data to provide guidelines for patients with type-2 diabetes	It enabled individual patients to avoid foods that cause blood glucose spikes and to replace them with foods that do not produce spikes.
2021	He et al. (65)	constructing a DT of the lumbar spine based on AI, data analytics, motion capture system, IK method, FEM.	They built a shape-performance integrated DT body to predict the biomechanical properties of real lumbar spine.
2021	Zohdi (68, 69)	Zohdi built a framework combined DT and machine-learning based on a genomic algorithm and coupled with simplified equations for the relationship between the particles and the fluid.	It was used to ascertain the placement and flow rates of multiple ventilation units, so as to optimal ventilation systems.
2021	Pilati et al. (70)	They developed a DT system for the vaccination process and tested it in a clinic.	It allowed a real-time simulation and creates a dynamic vaccination center, thus improving the efficiency of vaccination.
2022	Hernigou et al. (67)	They built a DT model based on CT and AI technology and improved the accuracy of the model to identified the orientation and evaluated the compensation of the axis of subtalar joints.	They minimized the inaccuracy of manual selection of anatomical landmarks by imaging systems.
2024	Hwang et al. (54)	A DT of the heart was built by CT and electroanatomical mapping data of the patient’s left atrium.	It is able to induce and observe virtual atrial fibrillation in virtual space and test the effects of various concentrations of virtual amiodarone after ablation.
2024	Jin et al. (55)	Integrating patient CT and electrophysiological mapping data to construct a highly simulated atrial fibrillation DT.	Simulated catheter ablation to compare the effects of different pulmonary venous space in the treatment of atrial fibrillation.
2024	Ložek et al. (57)	They constructed a right ventricular model of tetralogy of Fallot to simulate the ability of personalized CRT.	The Digital Twins help to identify candidates for RV-CRT as part of the lifetime management of tetralogy of Fallot.
2024	Swaitly et al. (63)	They promoted the application of digital twins in personalized dental care and used the U-Net architecture to automatically segment teeth directly from CBCT images.	It improved the accuracy of the generated 3D model and reduced the time required for segmentation.
2024	Guo et al. (65)	They used digital twin technology to build a dynamic monitoring model of upper limb force.	It ensured the accurate solution of muscle force and promotes the application of digital twins in rehabilitation medicine.
2025	Vanzella et al. (58)	They prepared a digital twin model to meet the challenges of cardiac rehabilitation caused by the COVID-19 pandemic.	It improved patient compliance and quality of life, but also need ongoing support.

AI, artificial intelligence; CT, computed tomography; DT, digital twin; FEM, finite element method; IK, inverse kinematics.

The application of digital twins in cardiovascular system primarily involves disease prediction, personalized treatment for cardiovascular diseases, treatment visualization, and planning (50–52). Cardiovascular digital twins enable close monitoring of an individual’s cardiovascular health by integrating clinical data, genetic information, and continuously monitored physiological and biochemical data. By combining sensor data with AI algorithms, a digital model of the heart can be constructed. This model can simulate potential cardiovascular issues and medication effects by applying various interference factors. It allows for accurate tracking of both the physical heart and its virtual counterpart, evaluating the health status, and continuously monitoring and providing feedback continuously (53). Hwang et al. created a digital twin of a patient’s left atrium using data from computed tomography (CT) scans and electroanatomical mapping (EAM) (54). They developed a virtual left atrial model to induce and observe virtual atrial fibrillation, testing the effects of different concentrations of virtual amiodarone after ablation, aiming to advance personalized healthcare. Jin et al. the likelihood of diseases such as heart failure and myocardial infarction (55). For integrated patient CT and electrophysiological data to construct a high-fidelity atrial fibrillation model. They simulated catheter ablation in a human atrial fibrillation digital twin and compared the effects of treating atrial fibrillation under different pulmonary vein gap conditions. Digital twins can simulate the outcomes of various virtual interventions under the same, repeatable conditions, without ethical concerns. During this process, physicians can continuously adjust and optimize treatment plans. High-risk procedures can be tested on digital twin models before being performed in real life, ensuring lower risk during actual operations (56). Ložek et al. constructed a right ventricular model of tetralogy of Fallot to simulate the ability of personalized CRT to improve ventricular function and efficiency, and realized personalized treatment decisions driven by digital twins (57). Vanzella et al. adopted a digital twin model to meet the challenges of cardiac rehabilitation caused by the COVID-19 pandemic, improving patient compliance and quality of life (58).

Furthermore, as an innovative simulation and analysis tool, digital twins can provide decision support and optimization for the accurate diagnosis, surgical planning, and postoperative rehabilitation of orthopedic diseases (59–61). Similar to their application in cardiovascular diseases, digital twins can create a virtual surgical environment where doctors can simulate surgical plans and predict risks, thereby reducing the likelihood of surgical complications and improving patient outcomes (62). Swaitly et al. promoted the application of digital twins in personalized dental care. They used the U-Net architecture to automatically segment teeth directly from CBCT images, improving the accuracy of the generated 3D model and reducing the time required for segmentation (63). Guo et al. used digital twin technology to build a dynamic monitoring model of upper limb force to achieve real-time interactive operation between physical space and digital space. This model ensured the accurate solution of muscle force and promotes the application of digital twins in the fields of human rehabilitation medicine and sports training (64). Due to the versatility and high fidelity of digital twins, they can simulate biological representations from a single cell to a complete human body. This is achieved through advanced computational algorithms and artificial intelligence, enabling the acquisition of simulated experimental data. As a result, digital twins can play a crucial role throughout various stages of new drug development, including preclinical research and clinical trials, significantly accelerating the pace of drug development and the time it takes for drugs to reach the market (51). By modeling populations using the physiological and biochemical indicators collected from early-stage data and recruited subjects, virtual models that meet clinical trial requirements can be created (65). While individual differences mean that digital twins cannot fully replace real subjects, they provide immense value in drug development. This process allows for multiple drug trials, enabling the observation of the metabolic processes of drugs within the body (66). In the future, the further development and application of digital twins in the healthcare sector may gradually reduce reliance on real subjects. It can play an even greater role in the precise diagnosis of various systemic diseases, personalized treatments, and full-cycle rehabilitation, leading to more efficient and effective healthcare solutions (67).

### 3.2 Application of digital twin in urban community development

**Figure 2:**
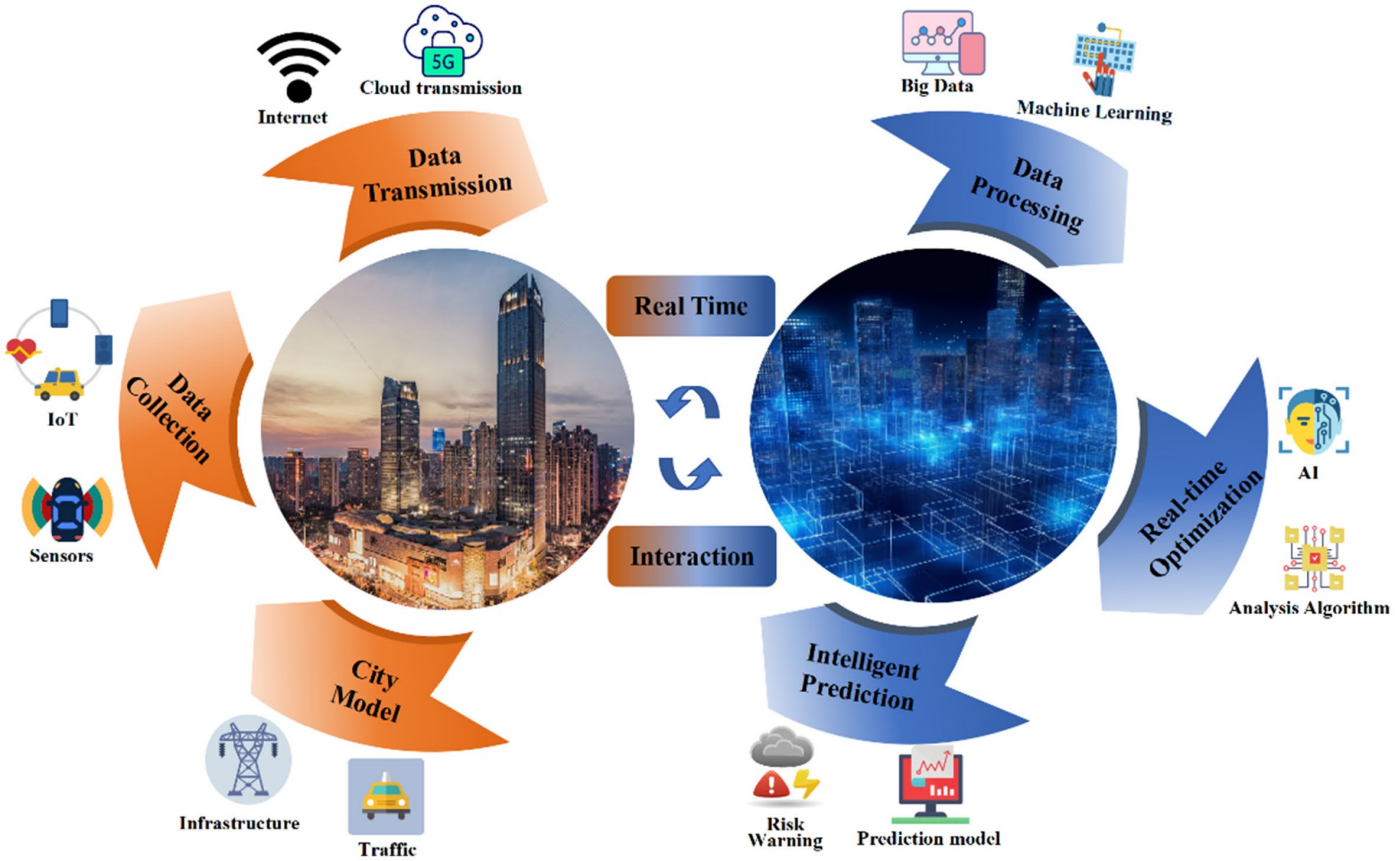
Application paradigm of digital twin technology in smart city.

In recent years, digital twins have become increasingly integrated into the construction of smart cities, giving rise to the concept of “digital twin cities.” By creating virtual replicas of urban environments and continuously evolving through learning, digital twins provide real-time insights into city planning through big data, machine learning, and artificial intelligence assistance (68). The application scenarios and models of digital twins in urban community development are illustrated in Figure 2. In the process of building and applying urban digital twins, IoT and sensor technology are used to collect data in urban physical space, transmit and preprocess it through the cloud, and build a multi-dimensional city model based on multi-source data. Collected data on infrastructure, traffic, weather, and other variables in digital space enable continuous monitoring of the city and predict its future development. This dynamic approach helps in optimizing urban planning, enhancing resource allocation, and improving the overall quality of life for city residents. Table 2 summarizes typical applications of digital twins in urban planning and development.

**Table 2:** Representative applications of DT in smart city.

Year	Team	Technical compositions of DT in smart city	The function of DTs in smart city
2022	Wolf et al. (74)	Develop a city digital twin based on Microsoft Azure cloud computing technology with integration of real-time weather and traffic data.	Providing a foundational framework for the construction and design of databases through urban digital twins for future smart city multi-agency incident response.
2022	Carles et al. (75)	Building 15-min city digital twin through open spatial data.	Barcelona’s urban accessibility was validated at the micro level based on the distribution of public facilities and services.
2022	Kuar (77, 78)	VIZZIO Technologies has deployed an extensive network of sensors, cameras and IoT devices throughout the city to collect real-time data.	The system has the ability to continuously monitor and adjust roads in real time to optimize traffic and improve urban mobility.
2020	Austin et al. (79)	Combining semantic knowledge representation and reasoning with machine learning to build digital city twin.	The system is being used to analyze the energy consumption of buildings in the Chicago area, which is important for smart city planning.
2022	Beckett et al. (80)	Integrating 3D modeling, visualization tools to build digital twin city.	Contribute to data-driven environmental planning and design to advance smart, sustainable urban development program.
2022	Zvarikova et al. (81)	Using urban sensing data to drive machine learning, integrated algorithms such as convolutional neural networks to build urban digital twin.	The framework enhances data-driven environmental planning processes to facilitate informed decision-making for sustainable urban development.
2023	De Raat et al. (82)	Amsterdam built digital twin through sensors and built the first 3D printed pedestrian bridge in history.	Data processing with 3D visualization was implemented in a geospatial environment for assessing service life.
2024	Gkontzis et al. (71)	Integration of AI technology to build a digital twin for Patras city, a coordinate-based problem mapping platform to capture citizen and city data.	Quickly understand the spatial distribution of citizen requests related to urban issues and predict the likelihood of issues recurring in the future.

AI, artificial intelligence; DT, digital twin; CNNs, Convolutional Neural Networks.

In recent years, the integration of AI technology with digital twin methodologies has significantly advanced the management and development of urban communities (69, 70). For instance, Gkontzis et al. combined AI with digital twin approaches to handle spatial issues in the interaction platform of Petros City citizens and urban data (71). Their framework quickly maps the spatial distribution of citizen requests related to urban problems based on specific coordinates. Additionally, the trained machine learning models in their framework predict the likelihood of recurring issues, thus enabling proactive urban management. In Los Angeles, the implementation of an intelligent traffic system leveraged digital twin simulations to test different traffic scenarios, optimizing signal lights and other infrastructure in real-time (72, 73). This system effectively addressed traffic congestion while enhancing the city’s mobility.

Wolf et al. developed a city digital twin using Microsoft’s Azure cloud computing technology (74). This model allows for the visualization of available resources, sends automatic updates, and integrates location-based real-time weather and traffic data. It serves as a foundational framework for the development and design of multi-agency event responses in future smart cities. Similarly, Barcelona has adopted an integrated smart city system with digital twin technology (75). By collecting real-time data on traffic conditions, available parking spaces, and bus usage, the system accurately monitors the status of the traffic network. Advanced analytics and AI algorithms applied to continuously updated digital twins help city workers precisely monitor the current traffic network and gain insights into traffic patterns, identify congestion hotspots, and forecast future mobility challenges. Similar applications have been successfully implemented in Chicago and Los Angeles, where the systems have been tested and validated (73, 76).

In Singapore, VIZZIO created the world’s largest digital twin—Virtual Singapore (77, 78). A wide array of sensors, cameras, and IoT devices are deployed throughout the city to collect real-time data from various urban systems. This data is analyzed using digital twin technology for accurate detection, intelligent decision-making, and improving the efficiency of public service departments. Austin et al. constructed a city digital twin to enhance data collection, event identification, and automated decision-making, emphasizing the importance of AI and machine learning in analyzing energy usage in Chicago (79). Beckett et al. explored an AI-driven, 3D modeling, visualization tool integrated with spatial cognition algorithms to provide a detailed understanding of evolving urban development patterns (80). Furthermore, Zvarikova et al. used different urban sensor data to drive machine learning models, creating a powerful and complex framework for enhancing the accuracy of spatiotemporal modeling in urban settings (81). Amsterdam has built the first 3D printed pedestrian bridge in history, and a digital twin has been created through sensors to track the performance of the structure under pressure in real time and predict the performance and life of the bridge (82).

It is noteworthy that digital twin cities can leverage data from past major crisis events to simulate emergency situations within a virtual city model. This simulation enhances the city’s ability to respond to sudden crises and increases its resilience. By preparing and planning for potential future challenges, digital twins provide a theoretical foundation and practical basis for the application of this technology in public health emergency management.

## 4 Digital twin technology in public health emergency management

As presented in the previous section, the concept of digital twin cities has demonstrated significant achievements in the digital transformation of urban environments, encompassing various sectors such as urban planning, resource management, and residential services. Meanwhile, the integration of real-time and static patient data into deep learning models, driven by advancements in digital healthcare, has laid a solid foundation for the application of digital twins in the medical field (31). Medical digital twins have demonstrated exceptional performance in areas such as healthcare data management, optimization of medical resource allocation, and innovation in healthcare services. In light of the sudden, unpredictable nature of public health emergencies, which often have widespread and severe consequences, we propose the development of a digital twin for urban public health emergency systems. By leveraging the PPRR (Prevention, Preparedness, Response, and Recovery) framework, this study analyzes the role of digital twin technology within public health management systems, exploring its potential to enhance efficiency and effectiveness in emergency management. The goal is to provide insights and recommendations for public health emergency response strategies, contributing to global public health security and offering decision-making support for the prevention and control of emergent health crises (83).

Unlike traditional “one-size-fits-all” emergency management strategies, the digital twin approach to public health emergency management integrates scientifically grounded disaster models to facilitate the customization of emergency response plans. In the DT-PPRR fusion framework, the value of digital twins comes from SF, MP, and DC. Meanwhile, they are subject to threshold adjustment from GC. They jointly conducted unified modeling and quantitative evaluation of the effect and value realization of digital twins in the PPRR stage. In the prevention stage, pay attention to early sentinel coverage and median data delay to measure SF, and characterize MP with early warning model drift detection rate and recalibration frequency to ensure that signals can be identified and corrected in time. DC is measured by risk communication reach rate and policy recommendation adoption rate, while GC is measured by cross-departmental data sharing agreement coverage and “minimum necessary” authorization achievement rate. In the preparation stage, SF is reflected in the completeness of the ledger of materials, beds and vehicle-connected equipment to ensure the availability and traceability of the data. MP is measured by the fit degree of historical data scenario database and the number of cross-scale interfaces, emphasizing multi-scenario compatibility. DC tests the linkage efficiency of each department by the average delay in the drill and the linkage system opening rate. GC is reflected in the audit closed-loop rate and interoperability compliance level, ensuring that the process is transparent and traceable. In the response phase, SF focuses on the perception and collection of emergency scene and clinical data to ensure the real-time and completeness of the data. MP is evaluated by online recalibration time-consuming and simulation-measured error (MAE/RMSE), which requires rapid fit to the actual situation. DC is measured by the scheduling execution binding rate and execution delay, and GC strikes the balance of compliance and delay through temporary authorization with contingency exemption. During the recovery phase, SF and MP adjust and update the model with follow-up data and review data, DC uses backlog clearing efficiency and policy implementation rate to describe preparation from response to normality, and transforming process experience into institutional normalization governance embodies GC.

**Figure 3:**
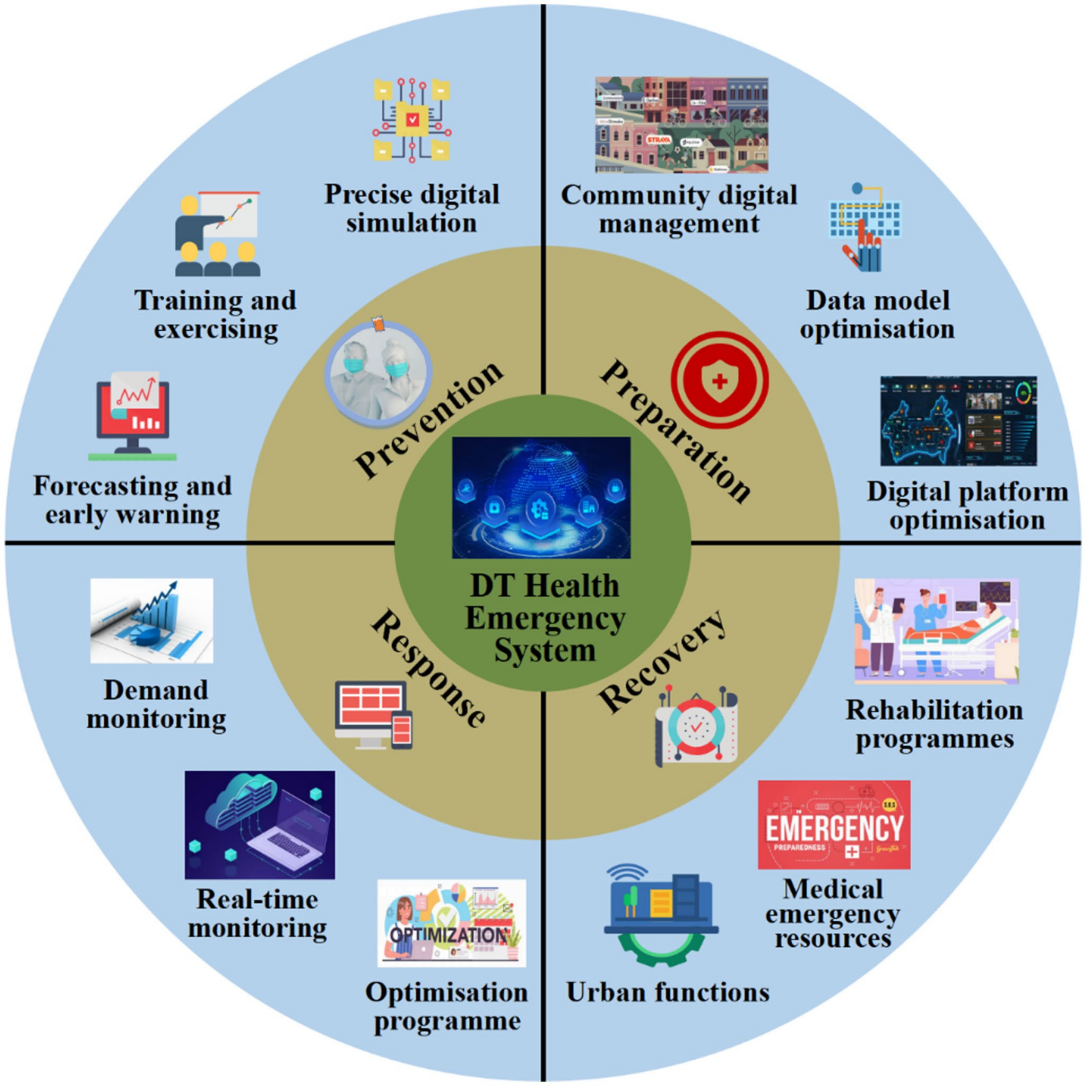
Implementation possibilities of digital twin technology in health emergency system, based on PPRR theory.

Digital twins can predict public health risks and assess the effectiveness of different response measures. As real-time monitoring or emergency event data are fed into the system, the digital twin is updated accordingly. In specific scenarios, the model can dynamically update by retrieving data from monitoring devices in real time. Similar to applications in the industrial sector, such as weather forecasting based on physical mathematical models, public health emergency management also prioritizes the use of mechanism-based models (84, 85). This approach avoids the inaccuracies often associated with unconstrained data-driven models in new or unforeseen situations. Ultimately, digital twins in public health emergency management can provide decision-makers with data-driven insights, supporting the development of personalized emergency intervention plans. Herein, we propose a core framework for the construction of a digital twin for public health emergency management systems based on the PPRR theory. According to this theory, the digital twin system for public health emergency management is divided into the following four components (Figure 3).

### 4.1 Prevention (P)

The comprehensive preparatory phase in emergency management functions as the “reserve warehouse” for the entire emergency process. During the prevention phase, big data analytics are employed to examine data within urban and public health management models, predicting potential public health risks and enabling proactive preventive measures. For instance, public health facility placement can be planned based on trends in urban population mobility and the patterns of disease transmission. Furthermore, the system facilitates the execution of routine simulated emergency drills and training for emergency management personnel.

#### 4.1.1 Prediction and early warning

The monitoring and early warning phase is the “watchtower” of the emergency management process. Once the early warning monitoring system platform is established, it facilitates enhanced real-time surveillance of emerging public health incidents, enabling the immediate activation of the subsequent phase of emergency response. The Digital Twin system’s versatility and real-time capabilities are leveraged to optimize the smooth and efficient operation of infectious disease detection systems while enhancing the overall surveillance framework (86, 87). It facilitates real-time information sharing and feedback across departments, allowing for timely prediction and early warning of potential public health risks.

The formulation of an emergency response plan is predicated on a scientifically sound basis, with the plan itself being informed by comprehensive historical data, thus ensuring that public health emergency management methods and steps are well-supported. Furthermore, data from multiple disciplines—such as virology, epidemiology, and sociology—in addition to real-time data collected from sensors, are integrated to refine the emergency response plan. The integration of these diverse inputs is then consolidated, simulated, and optimized within the Digital Twin platform. The incorporation of a digital city model facilitates the assessment of disease transmission risks across diverse regions, the prediction of outbreak hotspots, and the provision of early warning signals. This proactive approach enables local public health authorities to prepare and implement preventive measures in advance.

#### 4.1.2 Drills and training

During the preparedness phase, Digital Twin technology is utilized to collect and analyze real-time data on emergent public health events, including influenza outbreaks, with regard to geographic distribution of cases and population infection rates. The integration of multi-source data enables the continuous updating of the Digital Twin model. This enables the simulation of the resources required for public health events of varying severity, thereby enabling proactive resource preparedness. Furthermore, it enhances the surge capacity of the healthcare system to meet the demand for medical care during public health emergencies when capacity peaks. The Digital Twin platform facilitates the establishment of a “seamless” surge capacity across institutions and regions. Leveraging smart technologies enhances surge detection capabilities, facilitating the mobilization of diverse stakeholders for coordinated responses. This, in turn, strengthens preparedness and responsiveness during the preparedness phase, allowing for the development of detailed emergency plans that can be continuously optimized.

### 4.2 Preparation (P)

During the preparation phase, digital twin technology is utilized to collect and analyze real-time data from public health emergencies, such as influenza outbreaks, including the geographic distribution of flu cases and infection rates within populations. By integrating data from multiple sources, the digital twin model is continuously updated. This enables the simulation of the resources required for public health events of varying severity, allowing for proactive resource allocation and preparedness. Additionally, the system enhances the surge capacity of the healthcare system, which refers to its ability to meet peak demand during emergencies. The digital twin platform facilitates the creation of a “seamless” surge capacity across institutions and regions, using intelligent technologies to improve surge recognition capabilities. It also mobilizes multiple stakeholders to respond collaboratively, thereby strengthening the emergency response capabilities during the preparation phase. Furthermore, detailed emergency response plans are developed and continuously optimized. This model can be used to monitor the progression of an epidemic and predict future trends, providing valuable data-driven insights for public health decision-making.

#### 4.2.1 Resource allocation and positioning

In the process of constructing a Digital Twin model for public health emergency resources, it is imperative to incorporate key resource elements, including but not limited to medical equipment, pharmaceuticals, and protective supplies. A comprehensive, real-time monitoring system is established for these resources. This is achieved through the implementation of a sophisticated network of sensors and advanced data transmission technologies, enabling the precise capture and recording of the operational status of each piece of medical equipment, the stock levels of pharmaceuticals, and the condition of each protective item. In the preparedness phase, the Digital Twin model facilitates the rapid and accurate identification of the location, operational status, and potential risks associated with each resource, thereby enabling optimized scheduling. Furthermore, emergency response plan models, which integrate medical and public health data, are digitized and stored in cloud databases. These models are simulated and optimized on the Digital Twin platform to ensure the feasibility and effectiveness of the emergency plans.

#### 4.2.2 Data modeling and optimization

Digital Twin technology has the potential to make a substantial contribution to the development and digitalization of detailed emergency response plans in the field of public health emergency management. The utilization of big data and real-time data facilitates the analysis and integration of epidemiological data from specific regions into the Digital Twin system. The system is designed to undergo continuous updates in accordance with environmental changes. The system’s capacity to simulate various epidemic scenarios facilitates the comprehension of virus mutations and transmission mechanisms, thereby enabling the implementation of preventive measures in advance. The system can also analyze the distribution of medical facilities and population density in urban areas, simulate epidemic spread, identify affected regions, and optimize the allocation of medical resources and emergency supplies. For instance, utilizing influenza models, the system can simulate the virus’s transmission path and mode, stages of spread, and susceptible populations, thereby facilitating a more profound comprehension of the virus’s dynamic and trends (88, 89). Furthermore, the integration of AI and sensor technologies has the potential to enhance the Digital Twin models for cities and public health emergency management. This facilitates the implementation of intelligent, community-based health emergency management, thereby maximizing the advantages of local communities and improving the efficiency and effectiveness of public health emergency responses.

### 4.3 Response (R)

In the event of a public health emergency, the emergency response phase functions as the “frontline” of the entire process. The Digital Twin system facilitates comprehensive, real-time monitoring of public health emergency conditions, extending from urban to community levels, based on live data. This encompasses the tracking of the velocity and extent of disease transmission, in addition to the efficacy of community containment measures. Concurrently, the system employs monitoring outcomes to expeditiously simulate, optimize, and adjust response strategies within the Digital Twin platform, thereby enhancing resource allocation. The enforcement of proportionate measures during emergency responses is of paramount importance in preventing the further escalation and worsening of the public health crisis. The implementation of well-coordinated, efficient, and evidence-based interventions is imperative to mitigate the impact of the emergency.

#### 4.3.1 Monitoring and calculating medical demand

In the aftermath of a public health emergency, the Digital Twin system has the capacity to expeditiously and precisely calculate the number of potential infections and the requisite medical resources in different regions and at various stages of the emergency. This is based on information such as disease transmission trends and the severity of the outbreak. The system is capable of determining the necessity for various medical resources, including but not limited to: medical equipment (e.g., ECMO, ventilators), pharmaceuticals, healthcare personnel, and protective gear. The integration of diverse data sources, encompassing residents’ health status, traffic flow, and utilization of public facilities, enables urban planners and public health experts to more effectively monitor the spread of infectious diseases. For instance, during a large-scale infectious disease outbreak, the Digital Twin system, through its predictive capabilities and calculations, combined with digital city models and pre-existing emergency resource reserve data, can precisely assess the status of transportation networks and the current distribution of medical resources. This facilitates the planning of optimal resource allocation pathways, ensuring that scarce medical resources are delivered to their destinations in a timely and accurate manner, even in emergency situations.

#### 4.3.2 Simulation of decision-making to derive the optimal solution

In the event of a public health emergency, the Digital Twin system can be utilized to simulate the entire patient journey, from registration, waiting, and diagnosis to follow-up, thereby optimizing the healthcare process. Concurrently, the system can gather real-time information from the field, including the scope of the epidemic, the number of infections, and the demand for medical resources. The Digital Twin system is designed to balance multiple factors, including economic, social, and healthcare environments, to select the optimal decision-making strategy and achieve the most efficient resource allocation.

### 4.4 Recovery (R)

The recovery phase functions as the “supply station” in the emergency management process. The proposed Digital Twin system in the recovery phase encompasses two dimensions: the recovery of patients’ health and the functional recovery of urban communities following a public health emergency. The utilization of the Digital Twin facilitates the evaluation of the progression and outcomes of the disease, the effectiveness of treatment methods, and the rationality of response measures. Furthermore, the system can predict the long-term impacts of public health emergency measures on urban society, the economy, and the environment, providing scientific evidence to guide the comprehensive recovery of cities.

#### 4.4.1 Patient disease recovery

The Digital Twin system has the capacity to integrate patients’ medical histories, vital signs, and dynamic changes during treatment, thereby enabling the creation of personalized rehabilitation plans tailored to individual patients in the context of public health emergencies. The utilization of visualization and virtual simulation capabilities within the Digital Twin framework facilitates the provision of remote rehabilitation guidance and health advice. This guidance is provided through a remote rehabilitation platform, offering real-time feedback and corrections based on the patient’s actual movements. The purpose of this real-time feedback is to ensure proper execution of rehabilitation exercises. Furthermore, the system’s capacity for analyzing extensive rehabilitation data sets and employing predictive modeling enables the forecasting of patients’ rehabilitation outcomes, providing early warnings about potential risks and challenges. This information can serve as a valuable reference for healthcare providers when developing subsequent treatment plans.

#### 4.4.2 Urban functional recovery

The Digital Twin system can quickly integrate various impact data related to a city’s response to and potential consequences from a public health emergency, including the extent of damage to medical facilities, disruptions to transportation systems, and the scope of stagnation in commercial activities. By analyzing these data, the system can accurately assess the degree of damage to urban functions, providing reliable information for subsequent recovery efforts. Additionally, by leveraging the Digital Twin of the city, the recovery process can be monitored dynamically, and policies can be adjusted in real time. This allows for continuous evaluation of the recovery progress of key urban indicators, as well as macro-level control of resource allocation and human capital. The system helps in formulating scientifically sound and rational recovery plans for urban functions. During the recovery phase, the Digital Twin system can also retrospectively review the progression of the public health emergency. By integrating data and simulating results, it can inform future urban public health planning. The data collected during the emergency response phase can be input into the system to provide valuable insights and references for future urban disease prevention and control efforts.

## 5 Discussion

The ultimate goal of digital twins in health emergency management is to create a system that mirrors the real public health system both in appearance and behavior, while also possessing the ability to predict future health emergencies. To achieve this goal, on the one hand, it is essential to integrate epidemiological data from public health emergencies with artificial intelligence algorithms to make data-driven scientific decisions. On the other hand, it is crucial to combine generalizable epidemiological data with real-time, patient-specific data to build models, which can then be operated and simulated on to better respond to various potential health emergencies.

This study aims to discuss the significant role and application value of digital twins in the public health domain. By constructing a comprehensive digital twin system for health emergencies, it enables multi-dimensional, real-time monitoring, simulation, and optimization. This, in turn, greatly improves the efficiency and effectiveness of health emergency management, providing a stronger and more reliable foundation for safeguarding public health. Utilizing the digital twin platform, a comprehensive emergency rescue framework can be developed, creating a multi-level rescue system, innovating social rescue methods, enhancing the efficiency of emergency response, and improving the timeliness and effectiveness of social aid. At the same time, by leveraging the timeliness and multi-source capabilities of the digital platform, an integrated mechanism for horizontal and vertical collaboration among multiple stakeholders can be established, clarifying the roles and responsibilities of various emergency response departments in real time.

### 5.1 The technical and practical challenges

When discussing the application of digital twin technology in public health emergency management, it is necessary to consider its technical and practical challenges in a comprehensive manner. At the technical level, the system relies on high computational power to process multidimensional heterogeneous data (e.g., population flow, virus transmission) in real time, but model complexity and network bandwidth constraints can cause computational delays, and regional digital divides (e.g., insufficient sensor coverage in underdeveloped areas) further undermine the accuracy of the twin model. In addition, idealized model assumptions (e.g., uniform population distribution) conflict with realistic spatio-temporal heterogeneity, and cross-sectoral data barriers hinder dynamic information integration. Limitations of the framework include sensitivity to data quality, high infrastructure costs, lack of scenario generalization, and system vulnerability to extreme events. In addition, although the conceptual framework proposed in this study provides a theoretical basis for follow-up research, it still needs to be combined with empirical data calculation and analysis in the future to provide a set of unified theoretical analysis templates and measurement paths for the field of health emergency.

In the future, hybrid modeling, resilient infrastructure design and cross-domain ethical governance are needed to facilitate the transformation of technology from theoretical validation to real-world resilience. Lastly, constructing a digital twin requires a large amount of data, but the accuracy of this data is crucial, as the reliability of the model can be easily affected by unreliable data. These limitations constrain the development of digital health emergencies. In future research, efforts can be made to establish unified operational standards, enhance data collection and processing technologies, improve the accuracy and effectiveness of data, and build more secure and reliable databases. These measures will help drive the development of digital twins in the broader public health field and promote the advancement of digital medicine.

### 5.2 Regional specificity

It is worth noting that the DT framework based on PPRR theory has been influenced by different economic levels and different political systems from construction to application. Among them, the economic level mainly affects SF and MP through resource endowment and the construction of digital infrastructure. Higher financial and information investment usually improves multi-source coverage and data freshness, and supports higher frequency model calibration and cross-scale coupling. Therefore, the marginal effects and differences reflected in health emergency DT in regions with different economic levels are most significant in the prevention/preparation stage. In addition, the institutional environment mainly affects DC and GC. Rule stability and cross-departmental collaboration maturity determine the execution receipt rate and execution delay. The rule of law and privacy compliance system, cross-departmental collaboration mechanism, performance and accountability rules, etc. Clarification and improvement are more conducive to creating higher GC in the response/recovery stage. In the future, on the basis of improving the framework quantification and evaluation system, quantitative indicators such as API penetration rate, execution binding rate, audit closed-loop rate, and interoperability standard adoption can be used to implement heterogeneity testing and weight reallocation, so as to quantify the economic impact SF/The role of MP and institutional impact DC/GC is quantitatively embedded in DT-PPRR evaluation and comparison.

### 5.3 Policy recommendations and trade-offs

It is important to note that digital twin technology is not a future technology. The successful application of digital twins in health emergency management will depend on the development of accurate risk assessment and intervention mathematical models based on a deep understanding of the fundamental mechanisms of public health emergencies. These types of digital twin systems can provide accurate risk prediction models, real-time data collection for model support, and effective interventions based on model outputs. Therefore, digital twins in health emergency management should not be viewed as replacements for existing emergency management methods, but rather as a tool to effectively translate ongoing foundational research into practical emergency responses. For government macro-coordination departments, it is necessary to formulate phased data sharing and temporary authorization rules for different PPRR scenarios, and clarify the “minimum necessary data set,” “use purpose restrictions” and post-audit to improve GC. At the same time, a unified interoperability standard is established to improve DC. MP and DC are improved by specifying regular drills, and the number and distribution of sentinels for real-time data collection are optimized to improve MP. For grassroots executive departments such as communities and hospitals, a digital twin sensing and scheduling plug-in for ICU/outpatient/emergency/operating room should be established, and a syndrome monitoring and community follow-up data recovery mechanism should be established. Lightweight IoT should be used to synchronize with batch processing to improve SF and MP. For data/communication technology supply departments, standardized SDKs for edge-cloud collaboration, data quality monitoring and integration blocks should be provided to support privacy-protected computing and improve SF/GC. In addition, legislative supervision and network information departments should clarify the exemption of data use for emergencies to improve GC.

Public health emergencies are highly uncertain and timely, so policy trade-offs need to be considered between the stability of the framework and emergencies to ensure the controlling of the system under when uncertainty surges. In the prevention/preparation stage, it is necessary to balance the early warning advance with the false alarm rate caused by early panic. In order to pursue a longer early warning advance, the risk of false alarm and induce emotional spillover may increase. A graded early warning and expert confirmation model should be established to avoid directly amplifying social media noise into public panic.

The response phase should balance the potential contradiction between digital twin-driven automated scheduling and audit/authorization by relevant departments. Rapid automated scheduling must be limited by event scope, use restrictions, temporary authorization whitelist and full-link audit, to achieve traceable, fast and legal calls between speed and compliance. At the same time, the response stage should also balance the efficiency of resource scheduling with the protection of vulnerable groups, improve the efficiency of medical resource scheduling in emergency events, and avoid allocation bias caused by a single efficiency orientation.

### 5.4 Data security and privacy

However, data security and privacy concerns are significant issues. In the public health field, digital twin technology involves large amounts of sensitive public health, medical, and personal privacy data. If this data were to be stolen by malicious actors, it could severely infringe on citizens’ right to life, health, and privacy. Issues related to data access and protection, regulatory approval, integrating data from different populations, and building trust with public health officials and communities during the decision-making process will turn the transformation of digital twins in health emergency management into a long-term battle, rather than a short-term sprint.

In this study, an integrated anonymization scheme is proposed under the framework: Under the principle of purpose limitation and minimum necessity, data classification and minimization are completed first. The direct identifiers such as name and age are removed and retention strategies such as purpose limitation and expiration destruction are set. Then, through the multi-layer technology superposition of table anonymization and differential privacy, privacy protection calculation and synthetic data, named entity recognition and desensitization processing are implemented on the data. At the governance level, establish pre-processing data protection impact assessment and audit, and clarify resharing prohibition and return/destruction clauses. The prevention and preparation phases prioritize aggregate release using minimal data sets, quasi-identifier generalization, and differential privacy. In the response phase, privacy computing is enabled under the constraints of the restrictions to support minute-level linkage. Minimize the risk of re-identification, and play a safe support role in DT-PPRR modeling, early warning and decision-making collaboration in sudden and normal scenarios.

## 6 Conclusion

Digital twin technology has already established a crucial application foundation in the medical field and the development of smart cities, making the construction of a digital twin system for health emergency management particularly critical. Based on digital twin technology and the PPRR framework, this study proposed strategies for constructing digital twins in the field of health emergencies and discussed their application value. A general analysis framework, composed of SF, MP, DC, and GC, was constructed as a theoretical tool to guide scheme design. Health emergency digital twins can realize the digitalization, intelligence, and visualization of the entire health emergency management process, thereby improve the efficiency and effectiveness of health emergency management and ensure public health and safety. In the future, it is necessary to consolidate the technical foundation and quantitative standards, and systematically test the above mechanisms and quantitative paradigms, aiming at making digital technology better applied in the field of public health.

## Data Availability

The original contributions presented in the study are included in the article/supplementary material, further inquiries can be directed to the corresponding author.

## References

[1] Clarke L Patouillard E Mirelman AJ Ho ZJM Edejer TT-T Kandel N. The costs of improving health emergency preparedness: a systematic review and analysis of multi-country studies. EClinicalMedicine. (2022) 44:101269. doi: 10.1016/j.eclinm.2021.101269 35146401 PMC8802087

[2] Peters DH Hanssen O Gutierrez J Abrahams J Nyenswah T. Financing common goods for health: core government functions in health emergency and disaster risk management. Health Syst Reform. (2019) 5:307–21. doi: 10.1080/23288604.2019.1660104 31661356

[3] World Health Organization. Emergency response framework (ERF). Geneva, Switzerland: World Health Organization. (2017).

[4] Khan Y O’Sullivan T Brown A Tracey S Gibson J Généreux M. Public health emergency preparedness: a framework to promote resilience. BMC Public Health. (2018) 18:1–16. doi: 10.1186/s12889-018-6250-7 PMC6280369 30518348

[5] Hao F Xiao Q Chon K. COVID-19 and China’s hotel industry: impacts, a disaster management framework, and post-pandemic agenda. Int J Hosp Manag. (2020) 90:102636. doi: 10.1016/j.ijhm.2020.102636, PMID: 32834356 PMC7405826

[6] Krausz M Westenberg JN Vigo D Spence RT Ramsey D. Emergency response to COVID-19 in Canada: platform development and implementation for eHealth in crisis management. JMIR Public Health Surveill. (2020) 6:e18995. doi: 10.2196/18995 32401218 PMC7236607

[7] Andres L Bryson JR Ersoy A Reardon L. Fragmented recoveries and proactive adaptability: new paradigm shifts, and theoretical directions to unpacking recovery processes and behavioural change In: Pandemic recovery?, Cheltenham, England: Edward Elgar Publishing (2024). 359–81.

[8] Yang Y Vinayavekhin S Phaal R O’Sullivan E Leelawat N. Strategic roadmapping framework for disaster response: case of COVID-19 pandemic vaccine rollout program in the UK. J Disaster Res. (2023) 18:11–20. doi: 10.20965/jdr.2023.p0011

[9] Chen S Zhang Z Yang J Wang J Zhai X Bärnighausen T. Fangcang shelter hospitals: a novel concept for responding to public health emergencies. Lancet. (2020) 395:1305–14. doi: 10.1016/S0140-6736(20)30744-3, PMID: 32247320 PMC7270591

[10] Zhang Y Cao X Wang P Wang G Lei G Shou Z. Emotional "inflection point" in public health emergencies with the 2019 new coronavirus pneumonia (NCP) in China. J Affect Disord. (2020) 276:797–803. doi: 10.1016/j.jad.2020.07.097, PMID: 32738664 PMC7369017

[11] Singh M Fuenmayor E Hinchy EP Qiao Y Murray N Devine D. Digital twin: origin to future. Appl Syst Innov. (2021) 4:36. doi: 10.3390/asi4020036

[12] Tao F Zhang H Liu A Nee AYC. Digital twin in industry: state-of-the-art. IEEE Trans Ind Inf. (2018) 15:2405–15. doi: 10.1109/TII.2018.2873186

[13] Groombridge D. Gartner top 10 strategic technology trends for 2023, Stamford. (2022).

[14] Javaid M Haleem A Suman R. Digital twin applications toward industry 4.0: a review. Cogn Robot. (2023) 3:71–92. doi: 10.1016/j.cogr.2023.04.003

[15] Mylonas G Kalogeras A Kalogeras G Anagnostopoulos C Alexakos C Muñoz L. Digital twins from smart manufacturing to smart cities: a survey. IEEE Access. (2021) 9:143222–49. doi: 10.1109/ACCESS.2021.3120843

[16] Lehtola VV Koeva M Elberink SO Raposo P Virtanen J-P Vahdatikhaki F. Digital twin of a city: review of technology serving city needs. Int J Appl Earth Obs Geoinf. (2022) 114:102915. doi: 10.1016/j.jag.2022.102915

[17] Tao F Zhang M Nee AYC. Digital twin driven smart manufacturing, Pittsburgh, America: Academic Press (2019).

[18] Kamel Boulos MN Zhang P. Digital twins: from personalised medicine to precision public health. J. Pers. Med. (2021) 11:745. doi: 10.3390/jpm11080745 34442389 PMC8401029

[19] Coorey G Figtree GA Fletcher DF Snelson VJ Vernon ST Winlaw D. The health digital twin to tackle cardiovascular disease—a review of an emerging interdisciplinary field. NPJ Digit Med. (2022) 5:126. doi: 10.1038/s41746-022-00640-7, PMID: 36028526 PMC9418270

[20] Sun T He X Li Z. Digital twin in healthcare: recent updates and challenges. Digit Health. (2023) 9:20552076221149651. doi: 10.1177/20552076221149651, PMID: 36636729 PMC9830576

[21] Xames MD Topcu TG. A systematic literature review of digital twin research for healthcare systems: research trends, gaps, and realization challenges. IEEE Access. (2024) 12:4099–126. doi: 10.1109/ACCESS.2023.3349379

[22] Katsoulakis E Wang Q Wu H Shahriyari L Fletcher R Liu J. Digital twins for health: a scoping review. NPJ Digit Med. (2024) 7:77. doi: 10.1038/s41746-024-01073-0, PMID: 38519626 PMC10960047

[23] Sun T He X Song X Shu L Li Z. The digital twin in medicine: a key to the future of healthcare? Front Med. (2022) 9:907066. doi: 10.3389/fmed.2022.907066, PMID: 35911407 PMC9330225

[24] Munagandla VB Pochu S Nersu S Kathram S. Real-time data integration for emergency response in healthcare systems. J AI-Power Med Innov. (2024) 3:25–38. doi: 10.60087/Japmi.Vol.03.Issue.01.ID.002

[25] Manikyala A Gade PK Venkata SSMGN Asadullah A Kommineni HP. Emergency response planning: leveraging machine learning for real-time decision-making. Technol Manag Rev. (2021) 6:50–62.

[26] Laili Y Zhang L Yang G. Chapter 9- a comprehensive method for model credibility measurement In: Zhang L Zeigler BP Laili Y, editors. Model engineering for simulation. Beijing, China: Academic Press (2019). 189–207.

[27] Yilmaz L Liu B. Model credibility revisited: concepts and considerations for appropriate trust. J Simul. (2022) 16:312–25. doi: 10.1080/17477778.2020.1821587

[28] Fernández-Godino M.G. Review of multi-fidelity models. Advances in Computational Science and Engineering. (2016) 351–400. doi: 10.3934/acse.2023015

[29] Absi GN Mahadevan S. Multi-fidelity approach to dynamics model calibration. Mech Syst Signal Process. (2016) 68:189–206. doi: 10.1016/j.ymssp.2015.07.019

[30] Stenseth NC Schlatte R Liu X Pielke R Jr Li R Chen B. How to avoid a local epidemic becoming a global pandemic. Proc Natl Acad Sci USA. (2023) 120:e2220080120. doi: 10.1073/pnas.2220080120, PMID: 36848570 PMC10013804

[31] Barricelli BR Casiraghi E Fogli D. A survey on digital twin: definitions, characteristics, applications, and design implications. IEEE Access. (2019) 7:167653–71. doi: 10.1109/ACCESS.2019.2953499

[32] Wenger C. The oak or the reed: how resilience theories are translated into disaster management policies. Ecol Soc. (2017) 22. doi: 10.5751/ES-09491-220318

[33] Tao Y. Safety and emergency management system for the elderly based on big data In: International conference on applications and techniques in cyber security and intelligence: Springer (2021) doi: 10.1007/978-3-030-79200-8_122

[34] Grieves M Vickers J. Digital twin: mitigating unpredictable, undesirable emergent behavior in complex systems In: Transdisciplinary perspectives on complex systems: New findings and approaches. Cham: Springer International Publishing (2017). 85–113.

[35] Grieves MW. Digital twins: past, present, and future In: The digital twin: Springer (2023). 97–121. doi: 10.1007/978-3-031-21343-4_4

[36] Grieves M.W. Virtually intelligent product systems: digital and physical twins. Florida (2019).

[37] Phanden RK Sharma P Dubey A. A review on simulation in digital twin for aerospace, manufacturing and robotics. Mater Today Proc. (2021) 38:174–8. doi: 10.1016/j.matpr.2020.06.446

[38] Boschert S Rosen R. Digital twin—the simulation aspect In: Mechatronic futures: challenges and solutions for mechatronic systems and their designers. Cham: Springer International Publishing (2016). 59–74.

[39] Gazerani P. Intelligent digital twins for personalized migraine care. J Pers Med. (2023) 13:1255. doi: 10.3390/jpm13081255, PMID: 37623505 PMC10455577

[40] Grieves M. Digital twin: manufacturing excellence through virtual factory replication. White Paper. (2014) 1:1–7.

[41] Tao F Liu W Liu J Liu X Liu Q Qu T. Digital twin and its potential application exploration. Jisuanji Jicheng Zhizao Xitong/Comput Integr Manuf Syst. (2018) 24:1–18. doi: 10.13196/j.cims.2018.01.001

[42] Satyanarayanan M. The emergence of edge computing. Computer. (2017) 50:30–9. doi: 10.1109/MC.2017.9

[43] Akbarialiabad H Pasdar A Murrell DF. Digital twins in dermatology, current status, and the road ahead. NPJ Digit Med. (2024) 7:228. doi: 10.1038/s41746-024-01220-7, PMID: 39187568 PMC11347578

[44] Wang G Badal A Jia X Maltz JS Mueller K Myers KJ. Development of metaverse for intelligent healthcare. Nat Mach Intell. (2022) 4:922–9. doi: 10.1038/s42256-022-00549-6, PMID: 36935774 PMC10015955

[45] Duan L Da Xu L. Data analytics in industry 4.0: a survey. Inf Syst Front. (2021) 26:1–17. doi: 10.1007/s10796-021-10190-0 PMC8384093 34456613

[46] Wang B Li Z Xu Z Sun Z Tian K. Digital twin modeling for structural strength monitoring via transfer learning-based multi-source data fusion. Mech Syst Signal Process. (2023) 200:110625. doi: 10.1016/j.ymssp.2023.110625

[47] Xie X Moretti N Merino J Chang JY Pauwels P Parlikad AK. Enabling building digital twin: ontology-based information management framework for multi-source data integration. IOP Conf Ser Earth Environ Sci. (2022) 1101:092010. doi: 10.1088/1755-1315/1101/9/092010

[48] An J Chua CK Mironov V. Application of machine learning in 3D bioprinting: focus on development of big data and digital twin. Int J Bioprint. (2021) 7:342. doi: 10.18063/ijb.v7i1.342, PMID: 33585718 PMC7875058

[49] Mandolla C Petruzzelli AM Percoco G Urbinati A. Building a digital twin for additive manufacturing through the exploitation of blockchain: a case analysis of the aircraft industry. Comput Ind. (2019) 109:134–52. doi: 10.1016/j.compind.2019.04.011

[50] Niederer SA Plank G Chinchapatnam P Ginks M Lamata P Rhode KS. Length-dependent tension in the failing heart and the efficacy of cardiac resynchronization therapy. Cardiovasc Res. (2011) 89:336–43. doi: 10.1093/cvr/cvq318, PMID: 20952413

[51] Baillargeon B Rebelo N Fox DD Taylor RL Kuhl E. The living heart project: a robust and integrative simulator for human heart function. Eur J Mech A Solids. (2014) 48:38–47. doi: 10.1016/j.euromechsol.2014.04.001, PMID: 25267880 PMC4175454

[52] Prakosa A Arevalo HJ Deng D Boyle PM Nikolov PP Ashikaga H. Personalized virtual-heart technology for guiding the ablation of infarct-related ventricular tachycardia. Nat Biomed Eng. (2018) 2:732–40. doi: 10.1038/s41551-018-0282-2, PMID: 30847259 PMC6400313

[53] Sel K Osman D Zare F Masoumi Shahrbabak S Brattain L Hahn JO. Building digital twins for cardiovascular health: from principles to clinical impact. J Am Heart Assoc. (2024) 13:e031981. doi: 10.1161/JAHA.123.031981, PMID: 39087582 PMC11681439

[54] Hwang T Lim B Kwon OS Kim MH Kim D Park JW. Clinical usefulness of digital twin guided virtual amiodarone test in patients with atrial fibrillation ablation. NPJ Digit Med. (2024) 7:297. doi: 10.1038/s41746-024-01298-z, PMID: 39443659 PMC11499921

[55] Jin Z Hwang T Kim D Lim B Kwon OS Kim S. Anti- and pro-fibrillatory effects of pulmonary vein isolation gaps in human atrial fibrillation digital twins. NPJ Digit Med. (2024) 7:81. doi: 10.1038/s41746-024-01075-y, PMID: 38532181 PMC10966060

[56] Joy G Lopes LR Webber M Ardissino AM Wilson J Chan F. Electrophysiological characterization of subclinical and overt hypertrophic cardiomyopathy by magnetic resonance imaging-guided electrocardiography. J Am Coll Cardiol. (2024) 83:1042–55. doi: 10.1016/j.jacc.2024.01.006, PMID: 38385929 PMC10945386

[57] Ložek M Kovanda J Kubuš P Vrbík M Lhotská L Lumens J. How to assess and treat right ventricular electromechanical dyssynchrony in post-repair tetralogy of Fallot: insights from imaging, invasive studies, and computational modelling. Europace. (2024) 26. doi: 10.1093/europace/euae024, PMID: 38266248 PMC10838147

[58] Vanzella LM Cotie LM Flores-Hukom M Marzolini S Konidis R Ghisi GLd M. Patients' perceptions of hybrid and virtual-only care models during the cardiac rehabilitation patient journey: a qualitative study. J Cardiovasc Nurs. (2025) 40:E91–E100. doi: 10.1097/JCN.0000000000001076 38206327

[59] Sun T Wang J Suo M Liu X Huang H Zhang J. The digital twin: a potential solution for the personalized diagnosis and treatment of musculoskeletal system diseases. Bioengineering (Basel). (2023) 10:627. doi: 10.3390/bioengineering10060627, PMID: 37370558 PMC10295686

[60] He X Qiu Y Lai X Li Z Shu L Sun W. Towards a shape-performance integrated digital twin for lumbar spine analysis. Digital Twin. (2021) 1:8. doi: 10.12688/digitaltwin.17478.1

[61] Ahmadian H Mageswaran P Walter BA Blakaj DM Bourekas EC Mendel E. A digital twin for simulating the vertebroplasty procedure and its impact on mechanical stability of vertebra in cancer patients. Int J Numer Method Biomed Eng. (2022) 38:e3600. doi: 10.1002/cnm.3600, PMID: 35347880 PMC9287026

[62] Hernigou P Safar A Hernigou J Ferre B. Subtalar axis determined by combining digital twins and artificial intelligence: influence of the orientation of this axis for hindfoot compensation of varus and valgus knees. Int Orthop. (2022) 46:999–1007. doi: 10.1007/s00264-022-05311-6, PMID: 35138455

[63] Swaity A Elgarba BM Morgan N Ali S Shujaat S Borsci E. Deep learning driven segmentation of maxillary impacted canine on cone beam computed tomography images. Sci Rep. (2024) 14:369. doi: 10.1038/s41598-023-49613-0, PMID: 38172136 PMC10764895

[64] Guo Y Liu Y Sun W Yu S Han XJ Qu XH. Digital twin-driven dynamic monitoring system of the upper limb force. Comput Methods Biomech Biomed Engin. (2024) 27:1691–703. doi: 10.1080/10255842.2023.2254881, PMID: 37713212

[65] Saracco R. Digital twins: bridging physical space and cyberspace. Computer. (2019) 52:58–64. doi: 10.1109/MC.2019.2942803

[66] Erol T Mendi AF Doğan D. Digital transformation revolution with digital twin technology In: 2020 4th international symposium on multidisciplinary studies and innovative technologies (ISMSIT), Istanbul, Turkey: IEEE (2020)

[67] Thangaraj PM Benson SH Oikonomou EK Asselbergs FW Khera R. Cardiovascular care with digital twin technology in the era of generative artificial intelligence. Eur Heart J. (2024) 45:4808–21. doi: 10.1093/eurheartj/ehae619, PMID: 39322420 PMC11638093

[68] Sepasgozar SM. Digital twin and cities In: The Palgrave encyclopedia of urban and regional futures, Berlin/Heidelberg: Springer (2023). 426–32.

[69] Sharifi A Tarlani Beris A Sharifzadeh Javidi A Nouri M Gholizadeh Lonbar A Ahmadi M. Application of artificial intelligence in digital twin models for stormwater infrastructure systems in smart cities. Adv Eng Inform. (2024) 61:102485. doi: 10.1016/j.aei.2024.102485

[70] Fan C Zhang C Yahja A Mostafavi A. Disaster city digital twin: a vision for integrating artificial and human intelligence for disaster management. Int J Inf Manag. (2021) 56:102049. doi: 10.1016/j.ijinfomgt.2019.102049

[71] Gkontzis AF Kotsiantis S Feretzakis G Verykios VS. Enhancing urban resilience: smart city data analyses, forecasts, and digital twin techniques at the neighborhood level. Future Internet. (2024) 16:47. doi: 10.3390/fi16020047

[72] Faliagka E Christopoulou E Ringas D Politi T Kostis N Leonardos D. Trends in digital twin framework architectures for smart cities: a case study in smart mobility. Sensors. (2024) 24:1665. doi: 10.3390/s24051665, PMID: 38475201 PMC10934255

[73] Goldin M. Los Angeles pilots digital twin project to aid building decarbonization. (2022). Available online at: https://www.smartcitiesdive.com/news/los-angeles-cityzenith-digital-twin-building-decarbonization/624878/ (Accessed December 20, 2024).

[74] Wolf K Dawson RJ Mills JP Blythe P Morley J. Towards a digital twin for supporting multi-agency incident management in a smart city. Sci Rep. (2022) 12:16221. doi: 10.1038/s41598-022-20178-8, PMID: 36171329 PMC9519921

[75] Ferrer-Ortiz C Marquet O Mojica L Vich G. Barcelona under the 15-minute city lens: mapping the accessibility and proximity potential based on pedestrian travel times. Smart Cities. (2022) 5:146–61. doi: 10.3390/smartcities5010010

[76] Yu X. Merritt J. Digital twin cities: Framework and global practices. (2022). Available online at: https://www.weforum.org/publications/digital-twin-cities-framework-and-global-practices/?DAG=3&gad_source=1&gclid=CjwKCAiAs6sBhBmEiwA1Nl8s1qcW3TrvfyRCk68Uoe1z1ECgL-aJrGTxVT4_DxgzXvLV6t5TPCUxoC9v8QAvD_BwE

[77] Cavada M Tight MR Rogers CD. A smart city case study of Singapore—is Singapore truly smart? In: Smart city emergence, Amsterdam, The Netherlands: Elsevier (2019). 295–314.

[78] Kaur D. Singapore cloned to be world’s largest digital twin country. (2022). Available online at: https://techwireasia.com/2022/06/singapore-cloned-to-be-worlds-largest-digital-twin-country/

[79] Austin M Delgoshaei P Coelho M Heidarinejad M. Architecting smart city digital twins: combined semantic model and machine learning approach. J Manag Eng. (2020) 36:04020026. doi: 10.1061/(ASCE)ME.1943-5479.0000774

[80] Beckett S. Smart city digital twins, 3D modeling and visualization tools, and spatial cognition algorithms in artificial intelligence-based urban design and planning. Geopolit. Hist. Int. Relat. (2022) 14:123–38.

[81] Zvarikova K Horak J Downs S. Digital twin algorithms, smart city technologies, and 3D spatio-temporal simulations in virtual urban environments. Geopolit. Hist. Int. Relat. (2022) 14:139–54.

[82] De Raat G Bakker J Luiten G Paulissen J de Vogel B Scholten H. Predictive twin for steel bridge in The Netherlands In: Life-cycle of structures and infrastructure systems, Milan, Italy: CRC Press (2023). 1003–10.

[83] Stenseth NC Schlatte R Liu X Pielke R Jr Li R Chen B. How to avoid a local epidemic becoming a global pandemic. Proc Natl Acad Sci. (2023) 120:e2220080120 36848570 10.1073/pnas.2220080120 PMC10013804

[84] Liu H Liu Y. Construction of a medical resource sharing mechanism based on blockchain technology: evidence from the medical resource imbalance of China. Healthcare. (2021) 9:52. doi: 10.3390/healthcare9010052, PMID: 33418859 PMC7825101

[85] Yu T Sang P. The analysis of urban collaborative governance in public health emergencies with fuzzy theory based on BP algorithm. Sci Rep. (2024) 14:31427. doi: 10.1038/s41598-024-82966-8, PMID: 39732906 PMC11682215

[86] Manocha A Bhatia M Kumar G. Smart monitoring solution for dengue infection control: a digital twin-inspired approach. Comput Methods Prog Biomed. (2024) 257:108459. doi: 10.1016/j.cmpb.2024.108459, PMID: 39426139

[87] Zohdi T. A digital-twin and machine-learning framework for ventilation system optimization for capturing infectious disease respiratory emissions. Arch Comput Methods Eng. (2021) 28:4317–29. doi: 10.1007/s11831-021-09609-3 34108839 PMC8179093

[88] Laubenbacher R Sluka JP Glazier JA. Using digital twins in viral infection. Science. (2021) 371:1105–6. doi: 10.1126/science.abf3370, PMID: 33707255 PMC8170388

[89] Bilal S Zaatour W Alonso Otano Y Saha A Newcomb K Kim S. City SEIRcast: an agent-based city digital twin for pandemic analysis and simulation. Complex Intell Syst. (2025) 11:1–29. doi: 10.1007/s40747-024-01683-x

